# Cooling-Regulated Crystalline Structure for Enhanced High-Temperature Capacitive Energy Storage in PTFE Dielectric Films

**DOI:** 10.3390/polym18212663

**Published:** 2026-10-28

**Authors:** Jian-Yong Jiang, Jian Wang, Xin Zhen, Hui Zhang, Ming-Yue Ma

**Affiliations:** 1 Wuzhen Laboratory, Tongxiang 314500, China; zhui970626@163.com (H.Z.); ma_mingyue@126.com (M.-Y.M.); 2 School of Materials Science and Engineering, Wuhan University of Technology, Wuhan 430070, China; jessica-zhen@whut.edu.cn

**Keywords:** PTFE, crystalline structure, cooling process, high-temperature energy storage

## Abstract

Polytetrafluoroethylene (PTFE) possesses outstanding thermal stability, low dielectric loss and an excellent insulating property, showing significant application potential in high-performance energy storage film capacitors. However, the conventional natural cooling process after high-temperature heat treatment tends to induce large fibrous crystalline structures accompanied by pores and pits, which limits the energy storage performance of PTFE films at high temperatures. Herein, flexible PTFE films with different crystalline morphologies are prepared by solution casting. By optimizing the cooling process, particularly through ice water bath cooling, crystalline growth is effectively suppressed, leading to a refined, homogeneous and dense crystalline structure. Simulation results demonstrate that the optimized crystalline structure effectively mitigates local electric field distortions, heat accumulation and stress concentration to improve the overall electrical–thermal–mechanical response. Consequently, the PTFE film prepared by ice water bath cooling exhibits an enhanced energy density of 2.33 J/cm^3^ with an efficiency of 92% at 200 °C under an electric field of 480 MV/m. The present work suggests that regulating the crystalline structure through the cooling process is a simple and low-cost strategy for effectively enhancing the high temperature energy storage performance of polymer dielectric films.

## 1. Introduction

Polymer film dielectrics have been widely employed in electronic and electrical systems due to their significant advantages of rapid discharging rates, high power density, excellent processability and outstanding reliability [1,2]. With increasing device integration and the expansion of applications in extreme environments, the thermal loading on electronic devices becomes increasingly severe, imposing greater demands on the thermal stability and high-temperature performance of polymer dielectrics [3,4]. However, the current state-of-the-art commercial polymer dielectric, biaxially oriented polypropylene (BOPP), suffers from a rapid deterioration in energy storage performance when operating temperatures exceed 100 °C. Under elevated temperatures and high electric fields, thermally activated charge transport leads to increased leakage current and dielectric loss, which results in a significant decrease in breakdown strength, energy density and discharge efficiency. Consequently, developing polymer dielectric films capable of maintaining excellent energy storage performance at elevated temperatures has long been a major research focus in dielectric capacitor technology [5,6].

Among various engineering polymers, polytetrafluoroethylene (PTFE) has attracted considerable attention owing to its exceptional thermal stability, intrinsically low dielectric loss and excellent electrical insulation [7,8,9]. However, as a typical semi-crystalline polymer, the dielectric performance of PTFE is strongly governed by its crystalline morphology. Structural parameters such as crystallinity, size and shape, as well as the crystalline/amorphous interfaces [10,11,12,13,14,15,16,17], directly influence the carrier transport process, local physical field distribution and corresponding polarization/breakdown behavior. Particularly under high temperature environments, the electrical, thermal and mechanical mismatches between crystalline and amorphous regions become increasingly pronounced [18,19,20], giving rise to local electric field distortion, thermal localization and stress concentration, which eventually deteriorate energy storage performance. Therefore, the rational regulation of the crystalline morphology is crucial for optimizing the dielectric performance of PTFE films. To improve the high-temperature performance of polymer dielectrics, numerous approaches have been proposed, including filler incorporation [21,22,23,24], interface engineering [25,26,27], chemical crosslinking and multilayer structural design [28,29,30,31,32]. Although these strategies have demonstrated considerable improvements in energy storage properties, they typically rely on foreign components or complex structural constructions, which inevitably introduce interfacial defects and increase processing difficulties. In comparison, regulating crystallization through processing optimization provides a more intrinsic strategy for tailoring the crystalline morphology without changing the material composition [33,34]. Nevertheless, the underlying relationships among processing parameters, crystalline structure and energy storage performance have not yet been systematically clarified. In particular, how processing-induced the crystalline morphology governs the high-temperature energy storage performance of PTFE films remains poorly understood.

In this work, flexible PTFE films are prepared by solution casting, subsequently employing different cooling processes to regulate their crystalline morphology. It is found that ice water bath cooling enables the PTFE film to form a refined and uniform crystalline structure, significantly improving the electrical–thermal–mechanical response of the PTFE film. Eventually, the PTFE film achieves an energy storage density of 2.33 J/cm^3^ and an efficiency of 92% at 200 °C under an electric field of 480 MV/m. These results demonstrate that regulating the crystalline structure of PTFE through cooling process engineering provides an effective strategy for enhancing the high-temperature energy storage performance of polymer dielectric films.

## 2. Materials and Methods

### 2.1. Materials

The PTFE aqueous suspension (D210C) was purchased from Dakin Industries Co., Ltd., Osaka, Japan, characterized by a solid content of 59–61 wt%, a particle size of approximately 0.22–0.25 μm, a pH of 8.5–10.5, and a viscosity of less than 35 cP at 25 °C. The size of the PTFE spherical particles is around 200 nm. Triton X-100 was obtained from Aladdin Biochemical Technology Co., Ltd., Shanghai, China. The PI membrane with the thickness of 100 μm was purchased from Suzhou Youma Electronics Co., Ltd., Suzhou, China.

### 2.2. Preparation of the PTFE Films

**Figure 1:**
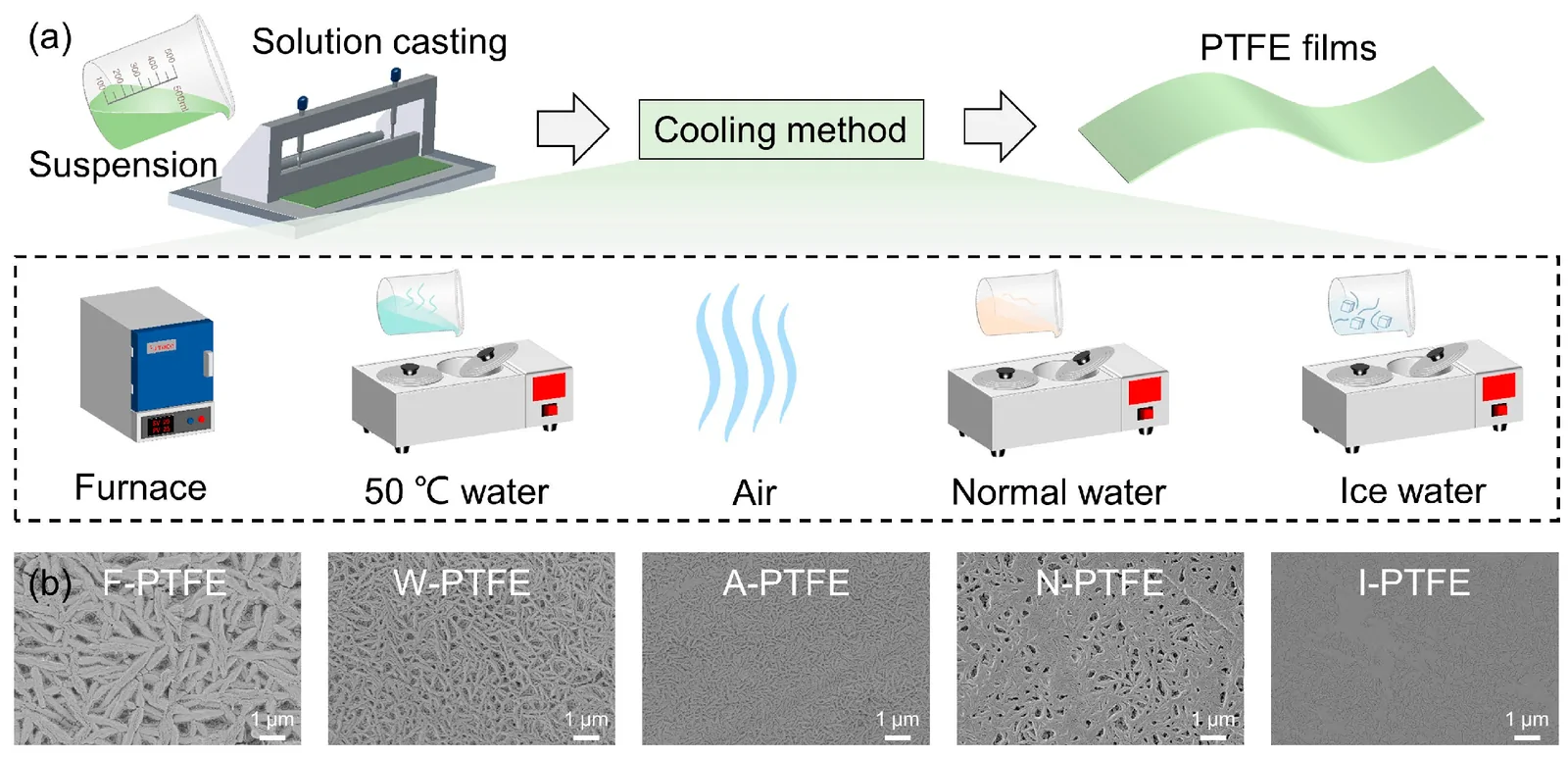
(**a**) Schematic diagrams of preparation processes of PTFE films with different cooling methods. (**b**) Surface SEM images of the prepared F-PTFE, W-PTFE, A-PTFE, N-PTFE, and I-PTFE films.

Figure 1a schematically illustrates the preparation process of PTFE films with different cooling methods. Firstly, 200 mL PTFE suspension was mixed in a flask and stirred at a speed of 200 r/min. As a nonionic surfactant, 5.27 mL Triton X-100 was taken and slowly dripped into the emulsion for about 8 ± 1 min to improve the dispersion stability and film-forming properties of PTFE aqueous dispersions. After dripping, the emulsion was devacuumized for 20 min. To prepare PTFE films, about 5 mL PTFE suspension was coated on the smooth surface of the PI base with the size of 10 × 10 cm. The scraper speed was 3 mm/s. The samples were dried at 45 °C in an oven for 1 h to remove the water. Then, the samples were transferred into a box furnace (KSL-1200X-M, HF-Kejing Co., Ltd., Hefei, China) for heat treatment. The temperature was increased from room temperature at 100 °C/h to 240 °C for 20 min, at which the surfactant contained in PTFE suspension was removed. The temperature was first increased to 350 °C at a heating rate of 60 °C/h to ensure sufficient plasticization throughout the samples. The heating rate was then increased to 100 °C/h until the temperature reached 380 °C, followed by holding at 380 °C for 40 min. Then the films were cooled and peeled off to study the influences of different cooling methods on the quality of the PTFE film. The cooling methods were as follows: furnace cooling, 50 °C water bath cooling, air cooling, normal temperature water bath cooling, and ice water bath cooling, denoted as F-PTFE, W-PTFE, A-PTFE, N-PTFE, I-PTFE, respectively. All samples were cooled from 380 °C for 20 min, and the temperature evolution during the cooling process was monitored using a thermocouple in close contact with the PTFE/PI film assembly (Figure S1). The thicknesses of PTFE films prepared in this study were about 6~8 μm (Figure S2).

### 2.3. Characterization

The morphology of PTFE films under different cooling processes was studied by scanning electron microscope (SEM, Sigma300, Carl Zeiss Microscopy Deutschland GmbH, Oberkochen, Germany). The X-ray diffraction pattern (XRD) was measured from 10° to 60° at the rate of 5°/min on a D8 Advance (Bruker AXS SE, Karlsruhe, Germany) employing Cu Kα radiation (*λ* = 0.154059 nm). Stress–strain curves were obtained at a tensile speed of 2 mm/min through a material testing machine UTM6502 (Suns Technology Stock Co., Ltd., Shenzhen, China) and PTFE films were cut into strip shapes (testing part with 15 mm width and about 0.008 mm thickness). For electric property measurement, top electrodes of Au were sputtered using a sputter coater (ZHD300, Beijing Technol Science Co., Ltd., Beijing, China) with a mask on the surface of the PTFE films, and the copper foil acted as the bottom electrode. The dielectric properties of the PTFE films were measured by an N5227B (Keysight Technologies, Inc., Santa Rosa, CA, USA) at the frequency range of 100 Hz~1 MHz. Electric displacement–electric field (*D-E*) loops were obtained at 10 Hz using a ferroelectric polarization tester (PK-CPE1901 PolyK Technologies , LLC, State College, PA, USA). In total, 10 samples used for breakdown were employed for each condition. The energy storage performances were calculated according to the *D-E* results.

### 2.4. Theoretical Simulations

Here, a comprehensive multi-physics field model has been developed to systematically investigate the coupled electrical, thermal, and mechanical responses of PTFE films. Based on the experimentally observed microstructure evolution, five crystalline/amorphous structures with different volume fractions and sizes of crystalline phases are designed. The local electric field, heat flux and stress distributions for different structures can be determined by solving the Poison’s equation, the heat conduction equation and the stress balance equation, respectively, which are described as

(1) $$\nabla (\varepsilon_{0}\varepsilon_{r}E(r,t))=0$$

(2) $$\kappa (r)\nabla T^{2}(r,t)=0,\;n\left(-\kappa \nabla T\right)=h\left(T-T_{\mathrm{sur}}\right)$$

(3) $$\nabla \varepsilon E_{Y}+F=0,\;F=\frac{1}{2}\varepsilon_{0}\varepsilon_{r}E^{2}(r,t)$$

where *ε*_0_, *ε*_r_, *E*, *κ*, *T*_sur_, *h*, *ε*, *E*_Y_ and *F* are the vacuum dielectric constant, dielectric constant, electric field, thermal conductivity, surrounding temperature, convective heat transfer coefficient, surrounding temperature, strain, Young’s modulus and Maxwell stress, respectively. Here, the *ε*_r_, *κ* and *E*_Y_ of the crystalline phase and the amorphous phase are set to 2.4, 0.3 W/(m·K) and 20 MPa and 1.6, 0.15 W/(m·K) and 6 MPa, respectively.

## 3. Results

### 3.1. Morphology and Crystallinity of PTFE Films

Figure 1b shows the surface SEM images of PTFE films prepared by different cooling methods, where it is observed that the microstructure of the PTFE films exhibits significant evolution as cooling conditions vary. The slow heat dissipation during the furnace cooling process provides substantial time for the migration and rearrangement of PTFE chain segments, as well as the continuous growth of crystalline regions. The prepared F-PTFE film exhibits a thick and fully grown fibrous crystalline structure, with numerous pores distributed among the overlapping crystallites, resulting in the relatively poor overall denseness of the film. Compared with F-PTFE, the fibrous crystalline phase structure in W-PTFE is significantly finer. As the cooling rate further increases (Figure S1), the size of the fibrous crystalline in A-PTFE and N-PTFE gradually decreases, accompanied by a significant reduction in the number of pores. When employing ice water bath rapid cooling, the film surface exhibits a nearly negligible accumulation of fibrous crystals and pore defects, presenting the most uniform and dense microstructure among all films. Therefore, the evolution of the microstructure demonstrates that rapid cooling effectively suppresses the extensive migration and rearrangement of PTFE chain segments and the excessive growth of crystalline phases, reducing crystalline size and inhibiting pore formation (Figure S3), ultimately resulting in a more uniformly dense microstructure.

**Figure 2:**
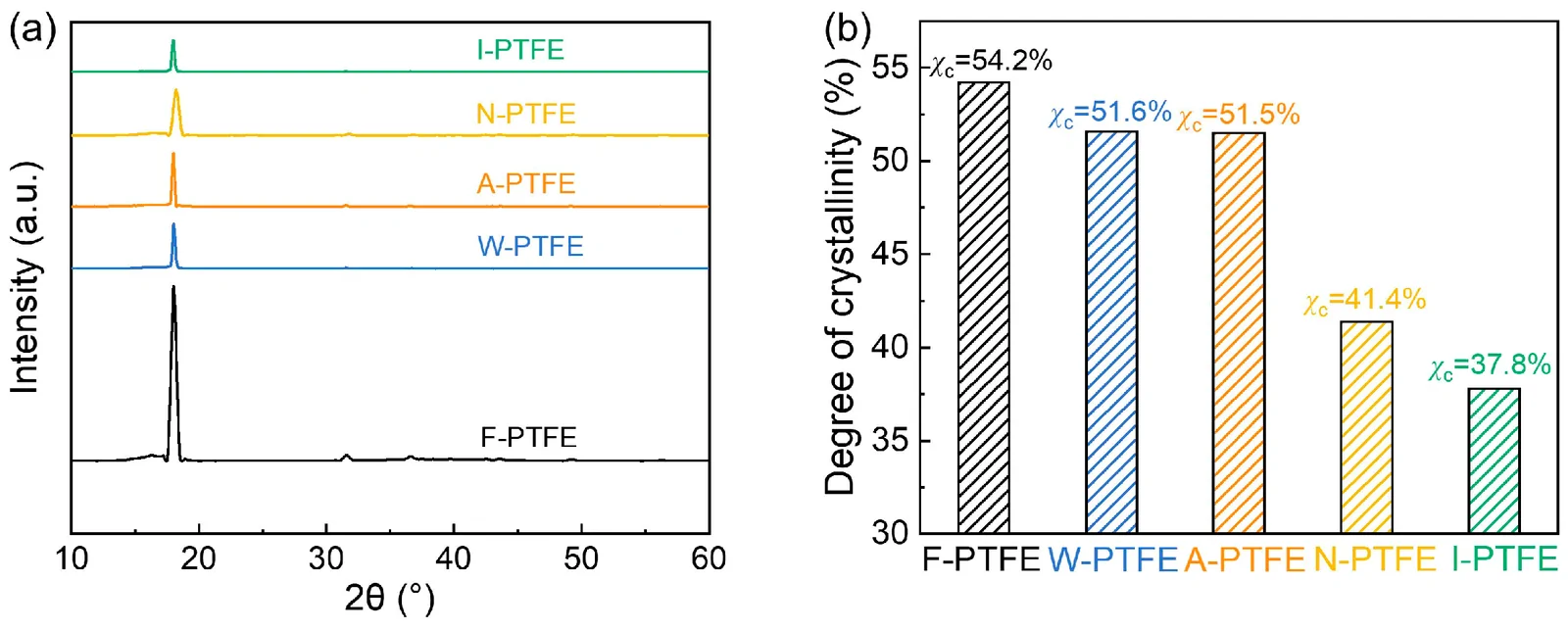
(**a**) XRD spectra and (**b**) degree of crystallinity of PTFE films with different cooling methods.

Furthermore, Figure 2 characterizes the crystalline behavior of PTFE films with different cooling methods. As shown in Figure 2a, all samples exhibit a distinct diffraction peak at 2*θ* of 18°, which corresponds to the characteristic (100) plane of crystalline PTFE. No new diffraction peaks or significant peak shifts are observed among the different samples, indicating that the change in the cooling process has not caused a phase transformation in PTFE. By comparison, there are significant differences in the intensities of the characteristic diffraction peaks among the samples, suggesting that cooling conditions primarily affect the degree of crystallinity in PTFE films. Through integration of the peak regions in the XRD patterns, the degree of crystallinity (*χ*_c_) of the different films is further estimated and shown in Figure 2b. The F-PTFE film exhibits a maximum *χ*_c_ of 54.2%, while the *χ*_c_ of W-PTFE and A-PTFE films decrease slightly to 51.6% and 51.5%, respectively. As the cooling rate increases, the *χ*_c_ of N-PTFE and I-PTFE decrease further and significantly to 41.4% and 37.8%, respectively. This variation primarily stems from the influence of differing cooling rates on the molecular chain rearrangement process. During slow cooling, the PTFE molecular chains have sufficient time to migrate and align, thereby promoting crystal growth and resulting in a high level of *χ*_c_. In contrast, as the cooling rate increases, the restricted movement of the polymer chains effectively inhibits the growth of crystalline phases, leading to the formation of more amorphous regions and a corresponding reduction in *χ*_c_. Furthermore, the reduced crystallinity weakens the constraints imposed by crystalline regions on amorphous chain segments, promoting the thermal motion and thereby affecting the corresponding glass transition behavior (Figure S4). Therefore, although rapid cooling reduces the overall crystallinity of the PTFE film, it effectively suppresses excessive crystal growth and the formation of pore defects, resulting in a more refined and homogeneous crystalline structure. This refined distribution of crystalline and amorphous regions helps to mitigate local electrical–thermal–mechanical mismatches, providing a favorable structural foundation for improving high-temperature capacitive energy storage performance.

### 3.2. Dielectric Properties of PTFE Films

**Figure 3:**
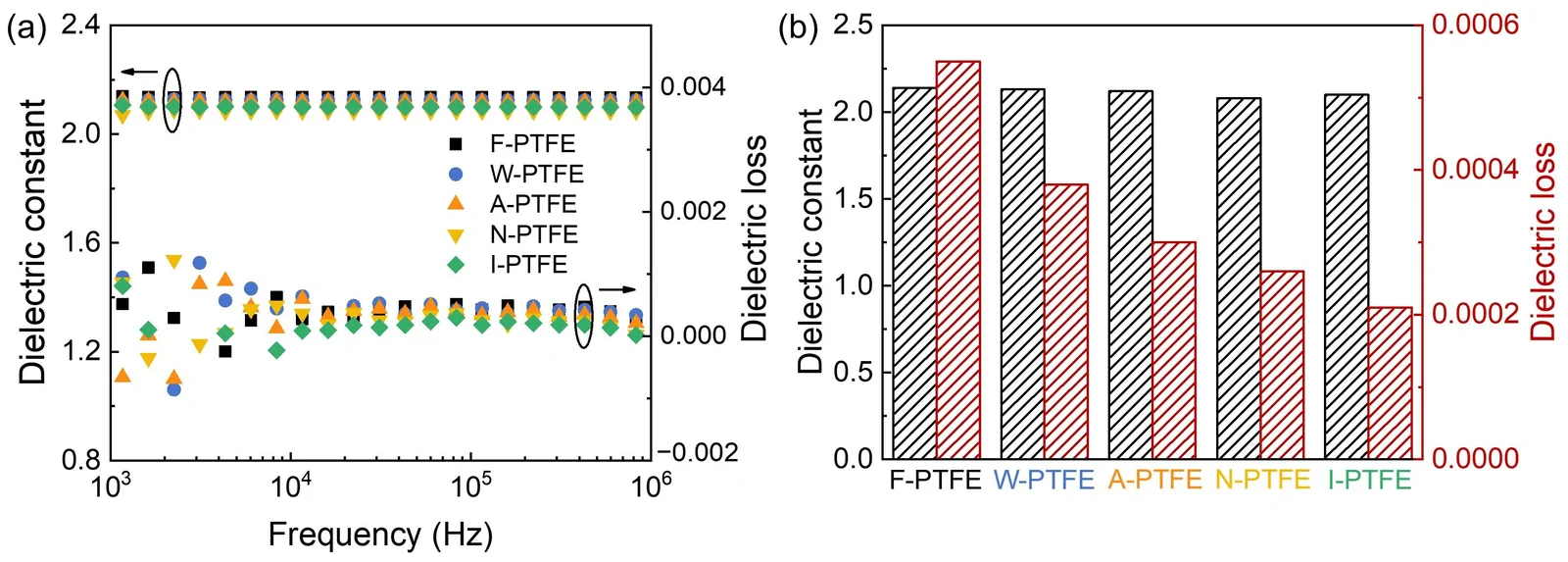
(**a**) Frequency dependence of dielectric constant and dielectric loss of PTFE films with different cooling methods at room temperature. (**b**) Dielectric constant and dielectric loss of PTFE films with different cooling methods at 10 kHz.

Firstly, the frequency dependence of the dielectric constant and dielectric loss of PTFE films with different cooling methods at room temperature is shown in Figure 3a. All PTFE films exhibit a dielectric constant of ~2.1 with negligible frequency dispersion over the frequency range from 10^3^ to 10^6^ Hz, indicating that varying the cooling process has little influence on the intrinsic polarization response of the PTFE film. In contrast, the dielectric loss is more sensitive to the cooling conditions. Although all films exhibit ultralow dielectric loss over the measured frequency range, a slight frequency dependence is observed at low frequencies before gradually reaching a stable value at higher frequencies. Figure 3b further summarizes the dielectric properties at 10 kHz. While the dielectric constants remain nearly unchanged, the dielectric loss decreases continuously from approximately 5.5 × 10^−4^ of F-PTFE to 2.1 × 10^−4^ of I-PTFE as the cooling rate increases. Furthermore, all PTFE films still exhibit stable dielectric constants and low dielectric loss at 150 °C and 200 °C, indicating their excellent high-temperature dielectric stability (Figure S5). The reduction in dielectric loss is closely related to the microstructure evolution induced by the cooling process. Rapid cooling suppresses excessive crystal growth and pore formation, resulting in a finer and denser microstructure. Such a structure significantly reduces defect-related charge transport and local electric field distortions, lowers dielectric loss and maintains the intrinsic dielectric constant of the PTFE film. Leakage current density measurements further confirm that increasing the cooling rate markedly suppresses electrical conduction (Figure S6), with I-PTFE exhibiting the lowest leakage current density over the entire electric field and temperature ranges. Therefore, the rapid cooling process enables a significant reduction in dielectric loss while maintaining the intrinsic polarization capability of PTFE. This synergistic combination of stable dielectric constant and low dielectric loss effectively reduces heating accumulation and energy loss under high temperatures and a high electric field, contributing to the enhanced high-temperature energy storage and efficiency of dielectric films.

### 3.3. Electrical–Thermal–Mechanical Simulations of PTFE Films

**Figure 4:**
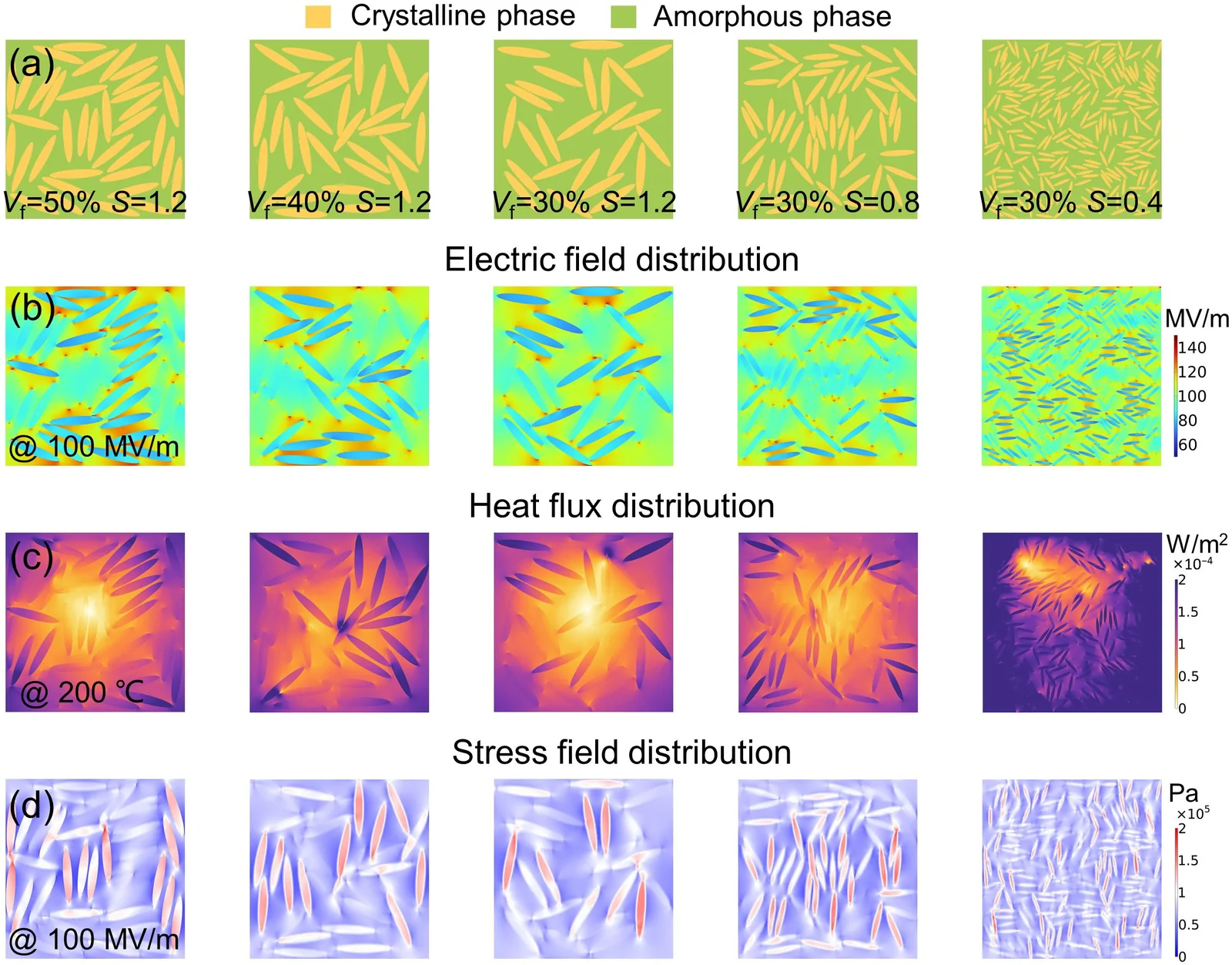
(**a**) Schematic diagram of PTFE films with different crystalline/amorphous composite structures. (**b**) Electric field distribution, (**c**) heat flux distribution and (**d**) stress field distribution of PTFE films.

To further understand how the cooling-regulated crystalline morphology influences the dielectric performance of PTFE films, representative crystalline/amorphous composite structures are established according to the experimentally observed microstructures for coupled electrical–thermal–mechanical simulations. As shown in Figure 4a, the fibrous crystalline phase (yellow region) is randomly embedded within the amorphous matrix (green region). Based on the experimentally observed evolution of the crystalline morphology, the representative crystalline volume fraction (*V*_f_) and the characteristic crystalline size (*S* = (*LW*)^1/2^, where *L* and *W* denote the long and short axes of the idealized fibrillar regions, respectively) are selected for the multiphysics simulations. These parameters are designed to represent the overall trends of decreasing crystallinity and progressive morphological refinement observed experimentally, rather than the exact microstructures of individual PTFE films. A controlled-variable approach was then employed to independently investigate the effects of *V*_f_ and *S* on the coupled electrical–thermal–mechanical response. Figure 4b presents the local electric field distributions under the applied electric field of 100 MV/m. Owing to the dielectric mismatch between the crystalline and amorphous phases, a composite structure with high *V*_f_ and large *S* exhibits significant localized electric field distortions near the crystalline/amorphous interface. As *V*_f_ and S decrease, the local electric field concentration weakens significantly, and the overall electric field distribution becomes more homogeneous, indicating that refining the crystal structure can effectively mitigate the electric field intensification at the interface. Additionally, the thermal response also exhibits a high dependence on the crystalline morphology, and the corresponding heat flux distributions at 200 °C are shown in Figure 4c. Owing to the discrete distribution of crystalline phases in the composite structure with high *V*_f_ and large *S*, discontinuous heat conduction paths are formed, resulting in a significantly inhomogeneous heat flux distribution and a tendency for local heat accumulation. With decreasing crystalline size and volume fraction, a refined and uniformly dispersed crystalline phase can generate a more efficient continuous heat transfer network, which enhances overall rapid heat dissipation. Consequently, the local heat accumulation is effectively alleviated, which is beneficial for improving the thermal stability of the PTFE films under elevated temperatures. Figure 4d further shows the local stress distributions of composites under the applied electric field of 100 MV/m; significant stress concentration is generated around the interfaces of large crystalline regions because of the mechanical incompatibility between the crystalline and amorphous phases. By contrast, the refined crystalline structure effectively homogenizes the stress distribution and suppresses interfacial stress concentration, which helps to reduce the risk of local mechanical failure. Additionally, the experimentally measured tensile properties show that the I-PTFE film also exhibits improved tensile strength and elongation at break (Figure S7), further confirming the beneficial effect of the refined crystalline morphology on mechanical performance. Therefore, the refinement and homogenization of the crystalline morphology simultaneously mitigate the local electric field distortion, heat accumulation and stress concentration. These synergistic improvements in the coupled electrical–thermal–mechanical response are expected to enhance the high temperature energy storage performance of PTFE films.

### 3.4. Electric Breakdown of PTFE Films

**Figure 5:**
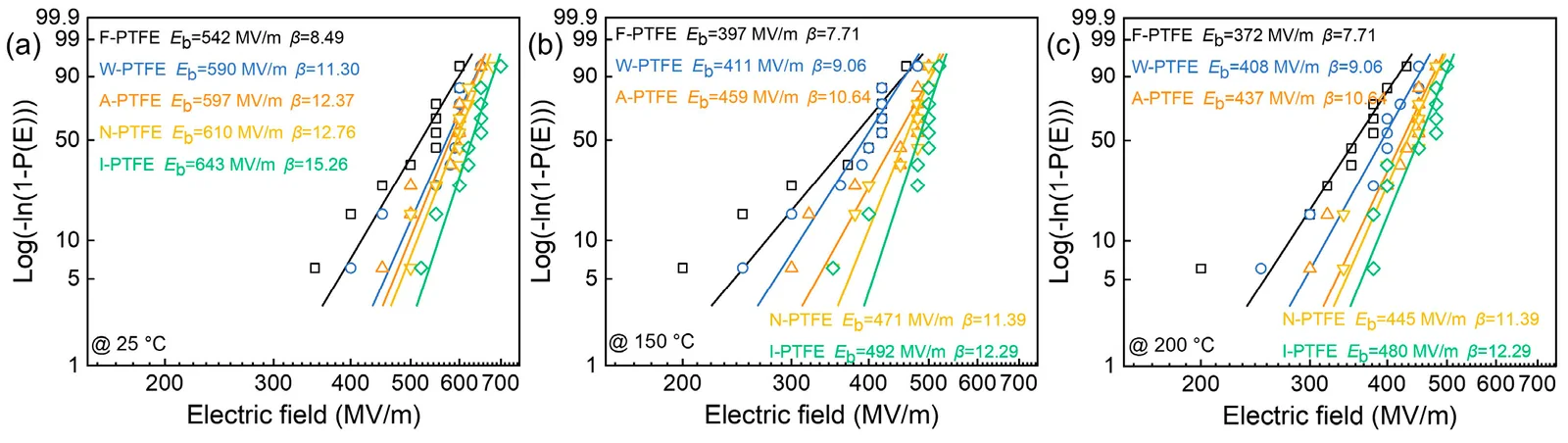
Weibull distributions of the breakdown strength of PTFE films with different cooling methods under various temperatures, (**a**) 25 °C, (**b**) 150 °C, and (**c**) 200 °C.

Then, the breakdown strength (*E*_b_) of the PTFE films is systematically evaluated, since it is one of the most critical parameters governing the energy storage capability of polymer dielectrics. Figure 5a–c show the Weibull breakdown distributions of PTFE films prepared by different cooling methods at 25 °C, 150 °C and 200 °C, respectively. For all samples, the breakdown strength decreases with increasing temperature (Figure S8). This behavior is mainly attributed to the thermally activated transport of charge carriers at high temperatures, which enhances electrical conduction and Joule heating, thereby intensifying the coupled electrical–thermal–mechanical degradation and accelerating breakdown failure. Nevertheless, the PTFE films prepared under rapid cooling consistently exhibit superior breakdown performance over the entire temperature range. In particular, the I-PTFE film achieves *E*_b_ of 643 MV/m, 492 MV/m and 480 MV/m at 25 °C, 150 °C and 200 °C, respectively, which are substantially higher than those of the other cooled films. Comparatively, the F-PTFE film exhibits the lowest *E*_b_, with the values of 542 MV/m, 397 MV/m and 372 MV/m at 25 °C, 150 °C and 200 °C, respectively. The enhancement in breakdown strength can be directly correlated with the cooling-regulated microstructure evolution. As discussed above, ice water bath rapid cooling suppresses excessive crystal growth and effectively reduces microscopic defects such as pores and pits, producing a refined and homogeneous crystalline morphology. Such a microstructure helps to optimize the electrical–thermal–mechanical response, thereby delaying the premature initiation of breakdown at high temperatures. Consequently, the rapidly cooled I-PTFE film exhibits significantly improved dielectric reliability, particularly under elevated temperature conditions.

### 3.5. Energy Storage Performance of PTFE Films

**Figure 6:**
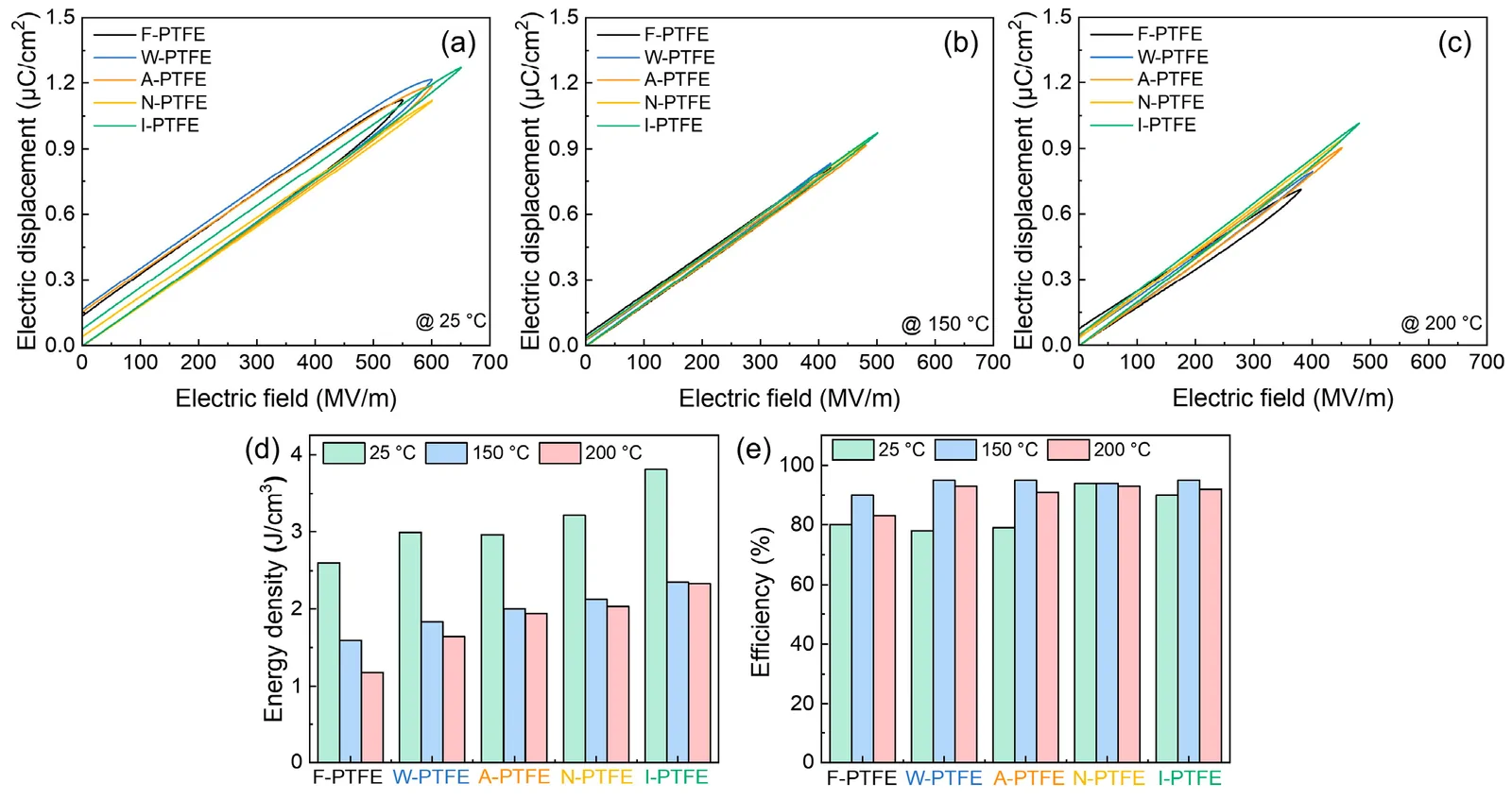
Comparison of displacement–electric field loops of PTFE films with different cooling methods under (**a**) 25 °C, (**b**) 150 °C and (**c**) 200 °C; comparison of (**d**) *U*_e_ and (**e**) *η* of PTFE films with different cooling methods under various temperatures.

To evaluate the energy storage performance of the PTFE films, Figure 6a–c show the unipolar displacement–electric field (*D-E*) loops of PTFE films prepared by different cooling methods at 25 °C, 150 °C and 200 °C. It can be seen that as the cooling rate increases, the PTFE film is able to withstand higher applied electric field and exhibits larger polarization. Among all the samples, the I-PTFE film exhibits a wider operating electric field window and a narrower *D-E* loop over the entire temperature range (Figures S9–S11), suggesting excellent energy storage characteristics. Then, the corresponding energy storage density (*U*_e_) and efficiency (*η*) calculated from the *D-E* loops are summarized in Figure 6d,e. Both the *U*_e_ and the *η* of films show continuous improvement trends as the cooling rate increased, with the I-PTFE film exhibiting the best energy storage performance at all temperatures (Figure S12). At 200 °C, I-PTFE achieved a *U*_e_ of 2.33 J/cm^3^ while maintaining a high *η* of 92%, which is 97%, 42%, 20% and 15% higher than F-PTFE (1.18 J/cm^3^), W-PTFE (1.64 J/cm^3^), A-PTFE (1.94 J/cm^3^) and N-PTFE (2.03 J/cm^3^), respectively. Moreover, I-PTFE exhibits good long-term electrical cycling stability at 200 °C (Figure S13), further demonstrating its potential for high-temperature capacitor applications. The enhancement in energy storage performance stems from the optimization of the microstructure induced by the rapid cooling process. The refined and homogeneous crystalline morphology can effectively improve its electrical–thermal–mechanical response, enabling the PTFE film to operate stably under higher electric fields while reducing energy loss, ultimately achieving a simultaneous increase in *U*_e_ and *η*. Overall, this work establishes a clear processing–structure–property relationship, demonstrating that cooling processing provides a simple yet effective route for tailoring crystalline morphology to enhance the high-temperature capacitive energy storage performance of the films.

## 4. Conclusions

In summary, the effects of the cooling process on the crystalline structure, coupled electrical–thermal–mechanical response and high-temperature energy storage performance of PTFE films are systematically investigated. The results indicate that as the cooling rate increases, the growth of crystals is effectively suppressed, resulting in a more refined and homogeneous crystalline structure with significantly reduced pores and defects. Multiphysics simulations further reveal that the optimized crystalline structure effectively mitigates local electric field distortions, heat accumulation and stress concentration, thereby improving the overall electrical–thermal–mechanical response and the dielectric reliability of the films under elevated temperatures. Consequently, the PTFE film prepared by ice water bath cooling achieved a breakdown strength of 480 MV/m at 200 °C, with an enhanced energy density of 2.33 J/cm^3^ and an efficiency of 92%, exhibiting excellent high temperature energy storage performance. This work demonstrates that crystalline engineering through cooling processes is a simple yet low-cost strategy for improving the high-temperature energy storage performance of films, offering new insights for the development of high-temperature polymer dielectric capacitors.

## Data Availability

The raw data supporting the conclusions of this article will be made available by the authors on request.

## Supplementary Materials

The following supporting information can be downloaded at: https://www.mdpi.com/article/10.3390/polym18212663/s1, Figure S1: (a) The temperature variations of PTFE films over time under different cooling conditions, (b) The cooling rate of different PTFE films. Figure S2: (a) cross-sectional SEM image and micrometer measurement of I-PTFE, (b) Average film thicknesses of different PTFE films. Figure S3: The characteristic width of the crystalline regions and pore fraction of PTFE films under different cooling conditions. Figure S4: The differential scanning calorimetry (DSC) measurements of different PTFE films. Figure S5: Frequency dependence of dielectric constant and dielectric loss of PTFE films with different cooling methods at 150 °C and 200 °C. Figure S6: The leakage current density of different PTFE films at 25 °C, 150 °C, and 200 °C. Figure S7 (a) Tensile stress-strain curves and (b) elongation at break and tensile strength of the different PTFE films. Figure S8: The Weibull breakdown strength (Eb) and shape parameter (β) of PTFE films with different cooling methods; Figure S9: *D-E* loops for (a) F-PTFE, (b) W-PTFE, (c) A-PTFE, (d) N-PTFE, and (e) I-PTFE film under different electric fields at 25 °C; Figure S10: *D-E* loops for (a) F-PTFE, (b) W-PTFE, (c) A-PTFE, (d) N-PTFE, and (e) I-PTFE film under different electric fields at 150 °C; Figure S11: *D-E* loops for (a) F-PTFE, (b) W-PTFE, (c) A-PTFE, (d) N-PTFE, and (e) I-PTFE film under different electric fields at 200 °C; Figure S12: Variation of the energy density and efficiency of the F-PTFE, W-PTFE, A-PTFE, N-PTFE and I-PTFE film with the electric field at 25 °C, 150 °C and 200 °C. Figure S13: (a) Long-term cycling stability of I-PTFE at 200 °C and 200 MV/m, (b) The *D-E* loops at 200 MV/m after different cycle numbers, each cycle involved heating the sample from 25 °C to 200 °C, holding it at 200 °C for one hour, and then cooling down to 25 °C.
