# Supplementary material for: Cooling-Regulated Crystalline Structure for Enhanced High-Temperature Capacitive Energy Storage in PTFE Dielectric Films
Source: Polymers (Basel). 2026 Oct 28;18(21):2663. doi: 10.3390/polym18212663 (PMC13765769; doi:10.3390/polym18212663)
Supplement: Supplementary file 1 [file polymers-18-02663-s001.zip › polymers-4626948-supplementary.pdf]

Supporting Information

Cooling-regulated crystalline structure for enhanced high-temperature capacitive energy storage in PTFE dielectric films

Jian-Yong Jiang[1,†,*], Jian Wang[2,†,*], Xin Zhen[2], Hui Zhang[1], Ming-Yue Ma[1]

[1] Wuzhen Laboratory, Tongxiang 314500, China

[2] State Key Laboratory of Advanced Technology for Materials Synthesis and Processing, Center of Smart Materials and Devices, Wuhan University of Technology, Wuhan 430070, China

[†] These authors contributed equally to this work.

*Correspondence: jiangjy@wuzhenlab.com (Jian-Yong Jiang), wang-jian@whut.edu.cn (Jian Wang)

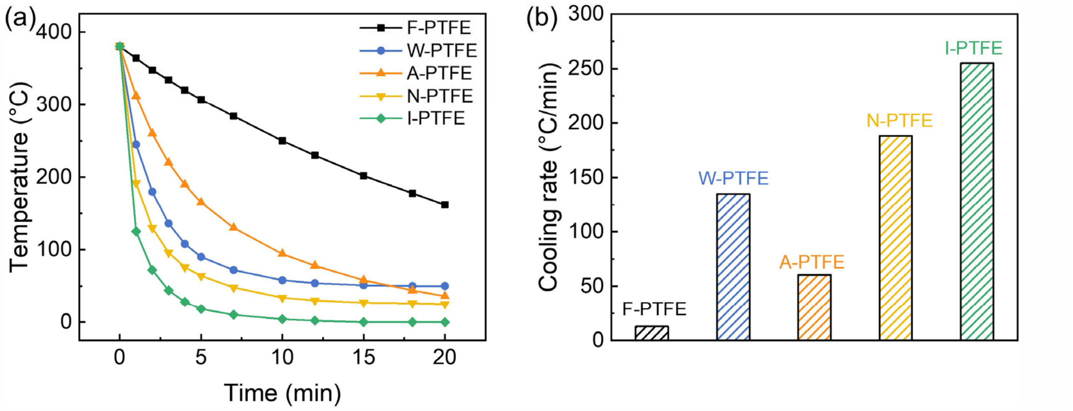

Figure S1. (**a**) The temperature variations of PTFE films over time under different cooling conditions, (**b**) The cooling rate of different PTFE films.

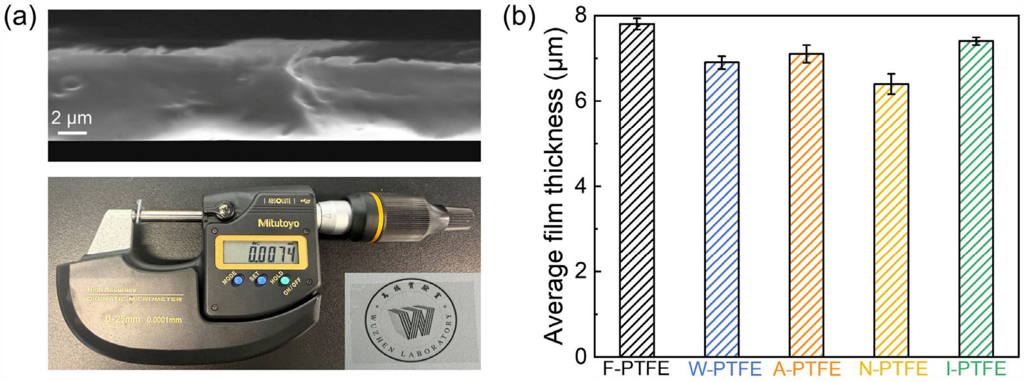

Figure S2. (**a**) cross-sectional SEM image and micrometer measurement of I-PTFE, (**b**) Average film thicknesses of different PTFE films.

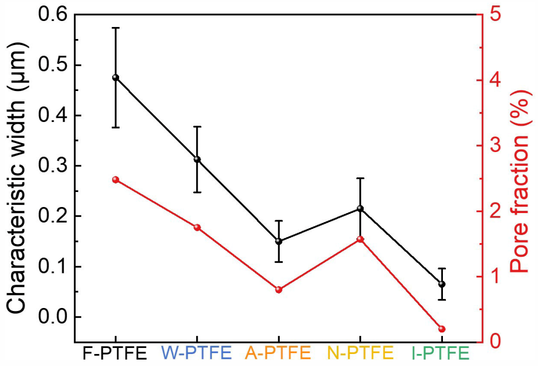

Figure S3. The characteristic width of the crystalline regions and pore fraction of PTFE films under different cooling conditions.

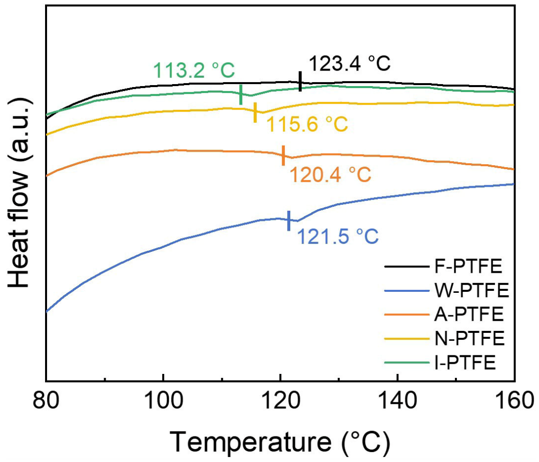

Figure S4 The differential scanning calorimetry (DSC) measurements o

f different PTFE films.

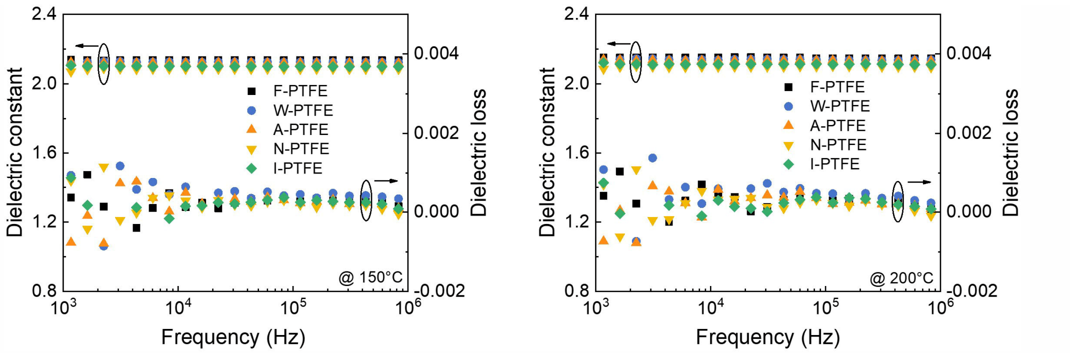

Figure S5. Frequency dependence of dielectric constant and dielectric loss of PTFE films with different cooling methods at 150 °C and 200 °C.

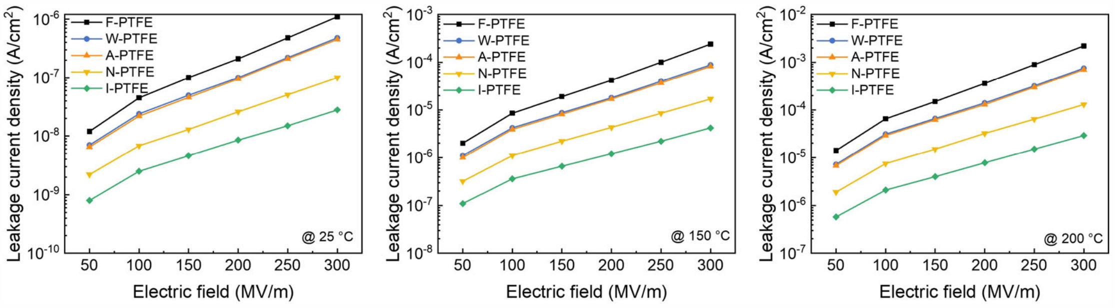

Figure S6. The leakage current density of different PTFE films at 25 °C, 150 °C, and 200 °C.

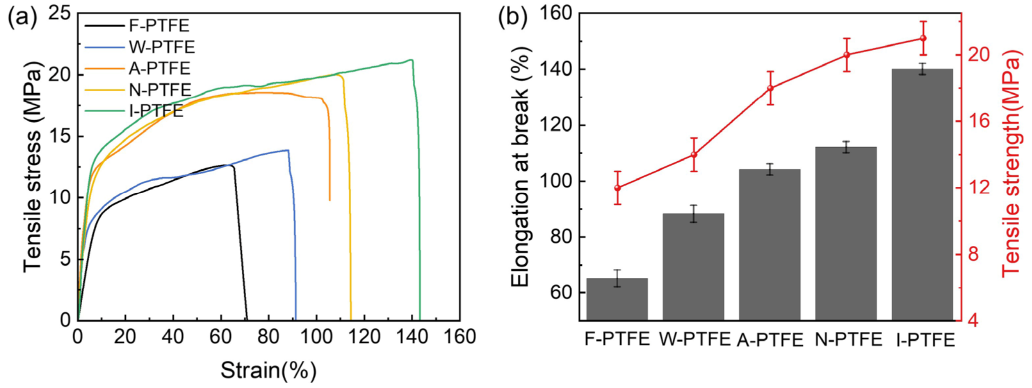

Figure S7 (**a**) Tensile stress-strain curves and (**b**) elongation at break and tensile strength of the different PTFE films.

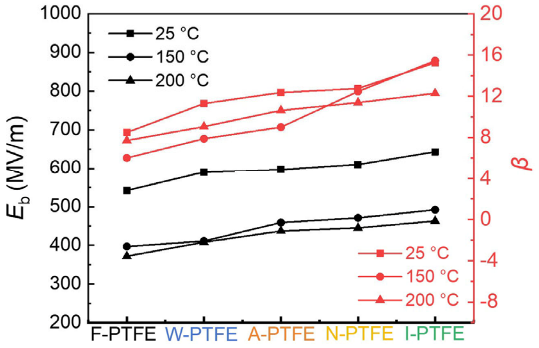

Figure S8. The Weibull breakdown strength (E_b) and shape parameter (β) of PTFE films with different cooling methods.

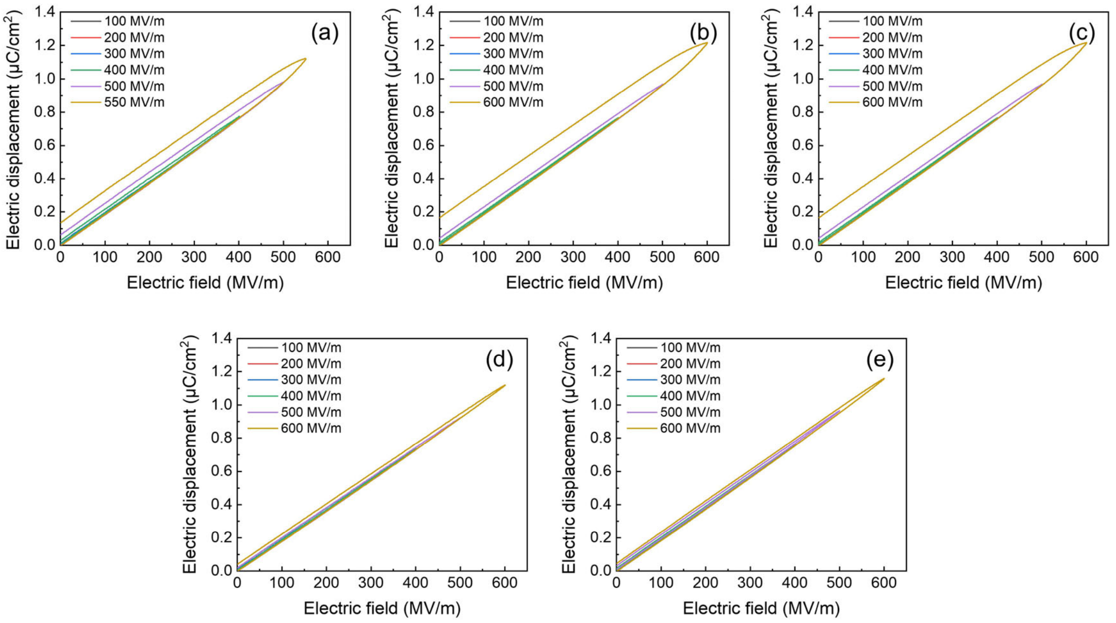

Figure S9. *D-E* loops for (a) F-PTFE, (b) W-PTFE, (c) A-PTFE, (d) N-PTFE, and (e) I-PTFE film under different electric fields at 25 °C.

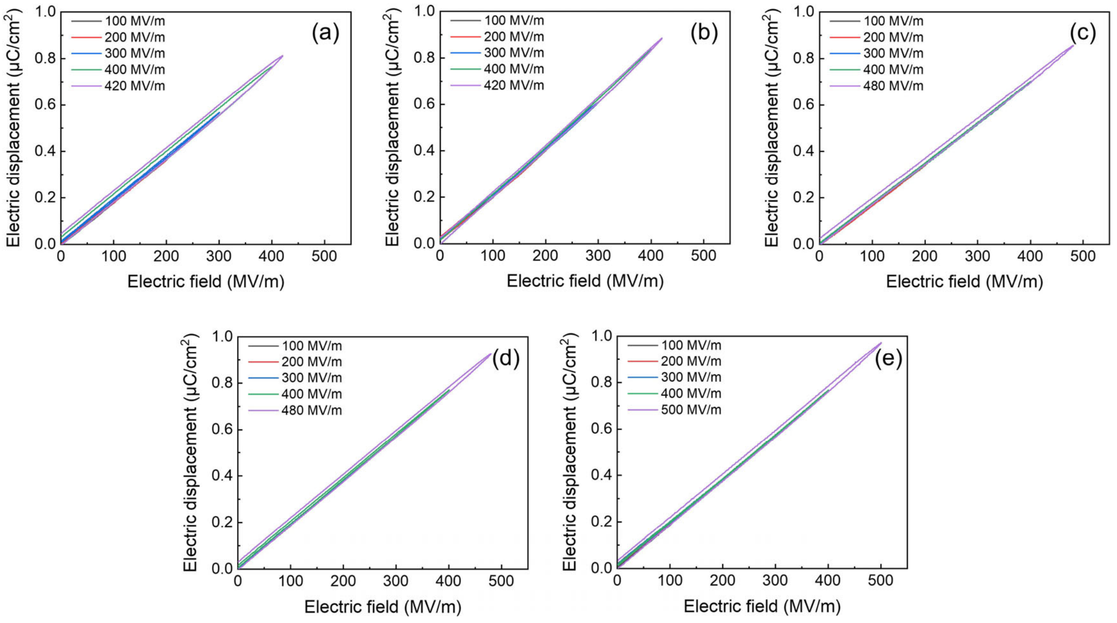

Figure S10. *D-E* loops for (a) F-PTFE, (b) W-PTFE, (c) A-PTFE, (d) N-PTFE, and (e) I-PTFE film under different electric fields at 150 °C.

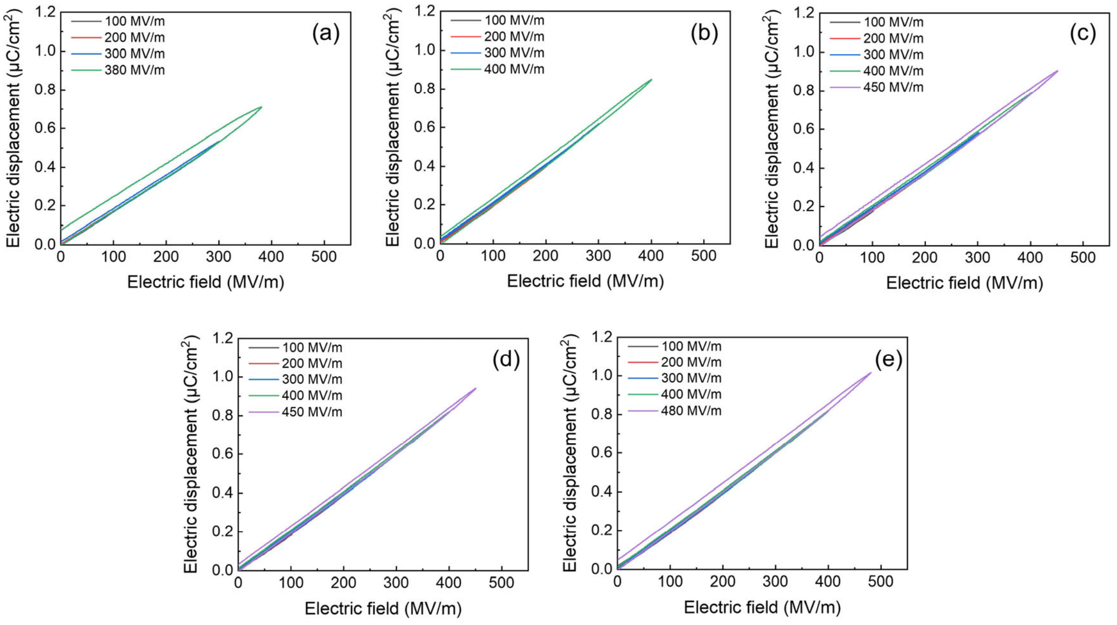

Figure S11. *D-E* loops for (a) F-PTFE, (b) W-PTFE, (c) A-PTFE, (d) N-PTFE, and (e) I-PTFE film under different electric fields at 200 °C.

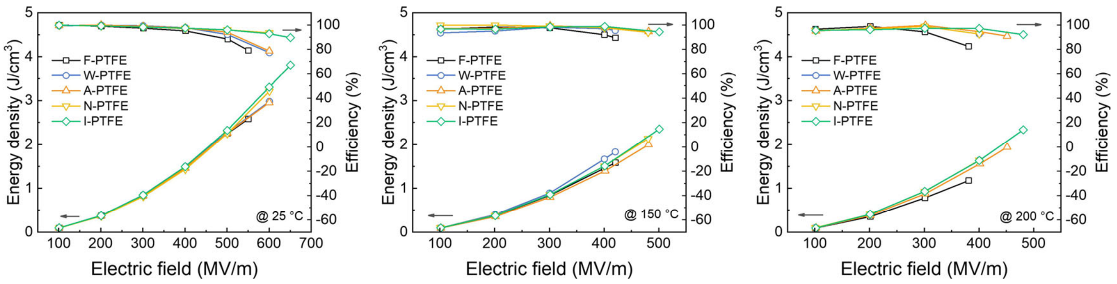

Figure S12. Variation of the energy density and efficiency of the F-PTFE, W-PTFE, A-PTFE, N-PTFE and I-PTFE film with the electric field at 25 °C, 150 °C and 200 °C.

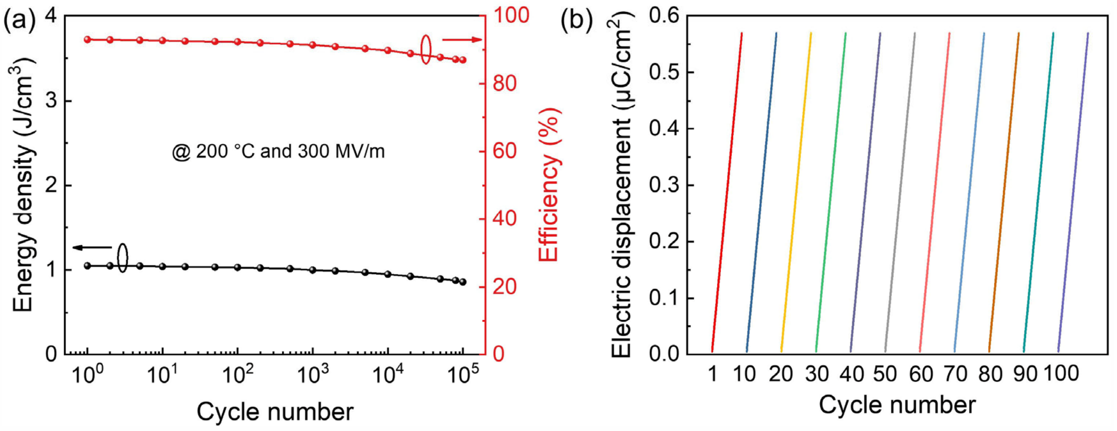

Figure S13. (**a**) Long-term cycling stability of I-PTFE at 200 °C and 200 MV/m, (**b**) The *D*-*E* loops at 200 MV/m after different cycle numbers, each cycle involved heating the sample from 25 °C to 200 °C, holding it at 200 °C for one hour, and then cooling down to 25 °C.
